# Multi-Angle Liquid Flow Measurement Using Ultrasonic Linear Array Transducer

**DOI:** 10.3390/s20020388

**Published:** 2020-01-10

**Authors:** Thi Huong Ly Nguyen, Suhyun Park

**Affiliations:** School of Electrical and Electronics Engineering, Chung-Ang University, Seoul 06974, Korea; lynguyen0908@cau.ac.kr

**Keywords:** ultrasonic flowmeter, array transducer, transit-time, multiple angular compensation

## Abstract

Most ultrasonic flowmeters utilize several wedge sensors for transmission and reception. Thus, the location and alignment of the sensors are critical factors that determine the performance of the ultrasonic flowmeter. In this study, we proposed an ultrasound liquid flowmeter utilizing a 128-element linear array transducer with a transmit delay control for varying the incidence angles of ultrasound wave transmission. The performance of the flowmeter was evaluated at flow rates of 0–50 L/min in a specially designed pipe system. Flow estimation was performed with the transit-time method using cross-correlation with phase zero-crossing for sub-sample estimation. While a single plane wave approach performed invasive electromagnetic measurements with only 74% accuracy as a reference, a multiple angular compensation method with 24 angles was proposed to increase the accuracy of measurements up to 93%. This study demonstrated the capability of the non-invasive single-sided ultrasonic flowmeter with a linear array transducer for liquid flow measurements in the metal pipe system.

## 1. Introduction

Flowmeters [1] have been utilized for liquid and gas flow rate measurements in industrial and medical fields. There are several types of liquid flowmeters, such as turbine [1], vortex [2], electromagnetic [3,4], and ultrasonic flowmeters [1,5]. The ultrasonic flowmeter can be applied for either invasive or non-invasive measurements with relatively easy installation [6,7]. Moreover, it has a high sensitivity to flow change [8,9]. Owing to these advantages, ultrasonic flowmeters are widely used for liquid flow measurements [10]. Doppler and transit-time flowmeters are two general types of ultrasonic flowmeters. In the Doppler ultrasonic flowmeter, ultrasound waves propagating through the fluid are reflected by particles in the liquid, such as small bubbles or solids moving with the flow. The frequency shift between the transmitted and reflected signals indicates the flow rate [1]. However, the accuracy of the Doppler ultrasonic flowmeter is limited by its dependence on the properties of the particles (e.g., size, concentration, distribution, and the relation between particles and fluid velocity) [11]. The second type is the transit-time ultrasonic flowmeter, which calculates the flow rate according to the differential transit time of the reflected ultrasonic waves between the downstream and upstream flow of the liquid [12,13,14]. The reflection of ultrasound waves is mainly due to the impedance mismatch between the pipe walls and the fluid. The Doppler ultrasonic flowmeter is preferable when there are scattering particles in the fluid. In contrast, the transit-time ultrasonic flowmeter is used for clean fluids flow or for a non-invasive approach, which can use the strong signal from the pipe walls [11,15,16].

The performance of the transit-time ultrasonic flowmeter can be degraded for various reasons, including the misalignment of transmitter and receiver sensors, irregular pipe surface conditions, errors in time difference estimation, and non-invasive (i.e., clamp-on or air-coupled) installation of transmitter and receiver sensors [13,17]. To measure the transit-time difference, several ultrasound sensors are installed on the pipe [18]. The single-sided measurement uses two or more sets of wedge-shaped ultrasound sensors on the same side of the pipe [17]. Ultrasound waves transmitted from wedge sensors propagate at an angle through the pipe wall and fluid, and then are reflected from the opposite pipe wall and received by the sensor. Although this angle can be determined by considering the specifications of the pipe and Snell’s law, the location and alignment of multiple transmitter and receiver sensors by choosing the proper wedge sensor angle are critical factors in designing flowmeters.

As employing more sensors with several wave traveling paths can increase the accuracy of the flowmeter, previous studies have tried to use array sensors for the flowmeter [19,20,21]. One study showed the feasibility of a combination of two-dimensional (2D) ultrasound phased array transducer (4 × 4 and 8 × 8 elements) and single transducers invasively installed in pitch-catch mode [19,20]. They used beam angle control for the transmission and reception of the array elements to increase the signal-to-noise of the ultrasound signals for gas flow measurements. Another study used an ultrasound linear array transducer (128 elements) to measure liquid flow non-invasively from a single side by tracking nylon powders inserted into the fluid using the Doppler mode [21]. Their system had a limited number of data channels (20 channels) without specific beam angle control for the transmission or reception of the array elements. Thus, the feasibility of array transducers for flowmeters was shown, but they were applied to specific cases. In this study, we aim to implement the single-sided non-invasive ultrasonic liquid flowmeter by fully utilizing the delay control and channel data from a one-dimensional (1D) ultrasound linear array transducer.

By utilizing the delay control of the ultrasound system, an array transducer can replace multiple wedge sensors. When the transmit delays for wave transmission are controlled, the incidence angle can be adaptively modified without varying the physical position and wedge angle of the transducer. Hence, both the transmission and reception of ultrasound waves can be achieved by one flat array transducer. Ultrasonic radio frequency (RF) signals from multiple receiver channels will include all possible reflective paths from the pipe wall. Therefore, ultrasonic signals from the appropriate transducer receiver sensors can be used to calculate the flow rate.

In this study, a single-sided non-invasive ultrasonic flowmeter is proposed for liquids flowing through a metal pipe to obtain more accurate and precise flow estimates. The flowmeter was implemented by utilizing the delay control function of an ultrasound imaging system and array sensors. Afterward, the performance was evaluated using the ultrasound data acquired from the specially designed pipe system. We performed a thorough analysis of the experimental results for non-invasive liquid flow measurements using an ultrasound array transducer. Multi-angle based liquid fluid measurements were also suggested for further improvement.

## 2. Materials and Methods

### 2.1. Experimental Setup

**Figure 1:**
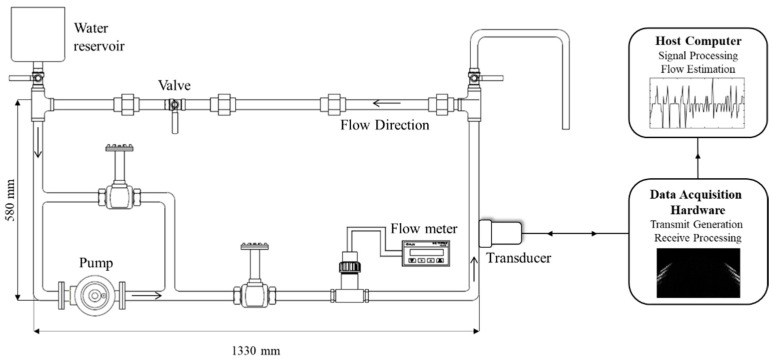
Experimental setup for liquid flow in the pipe system and ultrasound data acquisition system with array transducer.

Figure 1 shows the experimental setup for liquid flow measurements through the metal pipe using the ultrasound system with an ultrasound array transducer. Water in the pipe was circulated by a pump with a maximum capacity of 50 L/min. Additionally, the capacity of the water reservoir was 2 L. The pipe (SUS304) was made of carbon steel with an outer and inner diameter, as well as a wall thickness of 34 mm, 27.6 mm, and 3.2 mm, respectively. Flow rates, ranging from 0 to 50 L/min, were controlled by adjusting the pump (three flow modes at 0, 25, and 50 L/min) and valve of the pipe system for a subtle control between the fixed flow controls of the pump. By controlling the pump and valve of the pipe system, flow rates were adjusted for the experimental study at eight levels (approximately 0, 7, 10, 15, 20, 25, 35, and 50 L/min). An electromagnetic flowmeter (FMAG550G, Flos Korea Inc., Seoul, Korea) was installed through the pipe system invasively for real-time flow monitoring.

Two-dimensional (2D) ultrasonic signals were acquired by an ultrasound imaging system (Vantage 32LE™, Verasonics, Inc., Kirkland, WA, USA) with a linear array transducer (ATL L7-4, ATL Ultrasound Inc., Bothell, WA, USA). The ultrasound imaging system consists of a hardware for controlling wave transmission and data acquisition, and a host computer that controls all actions of the hardware and software environments using the Matlab software. Ultrasound waves were generated and received by the ultrasound imaging system with the linear array transducer. The system has 64 independently controlled transmitter channels and 32 receiver channels. The array transducer contains 128 elements with a frequency range of 4–7 MHz and a transducer size of 38 mm × 7 mm (i.e., width and height). The transducer transmission frequency was set at 5.2 MHz. The sampling frequency (*f*_s_) for system data acquisition was four times the transmission frequency. The transmitted signal was a pulsed wave with 2–3 cycles of sinusoidal waves to avoid any overlap between multiple reflections. The width of the transducer was aligned in the vertical direction of the pipe. Then, the height of the transducer was small enough to fit on the surface of the circular pipe (outer diameter is 34 mm). Thus, the flat linear transducer was coupled well on the pipe surface. Ultrasound gel was applied as a couplant agent to match the impedance between the pipe and the ultrasound transducer.

**Figure 2:**
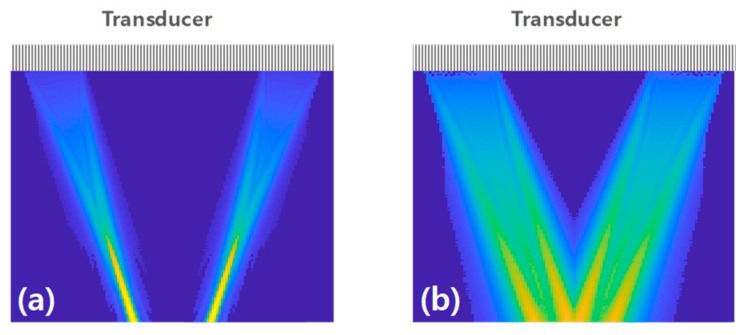
Transmitted ultrasound beam profiles of the (**a**) focused beam and (**b**) plane wave in the liquid. An array transducer was on top of the pipe.

A linear array transducer was utilized to alternately transmit and receive ultrasonic waves from both upstream and downstream of the liquid. The ultrasound beams were transmitted by activating 31 elements (i.e., elements 1–31 for downstream and elements 98–128 for upstream). In this study, two types of ultrasound waveforms were investigated: focused beam with a focal position at the surface of the pipe [22], and plane waves with no focus applied to the array transducer [23]. The incidence angle of the ultrasound path was 20.13°. The simulated transmitted beam profiles of the focused beam and plane waves are shown in Figure 2. It illustrates the active elements and the ultrasonic paths of the beams in the simulation of the beam transmission. All 128 elements were used to receive the reflected signals from the pipe. The received signals were transferred to the host computer and used for flow estimation.

### 2.2. Flow Estimation

**Figure 3:**
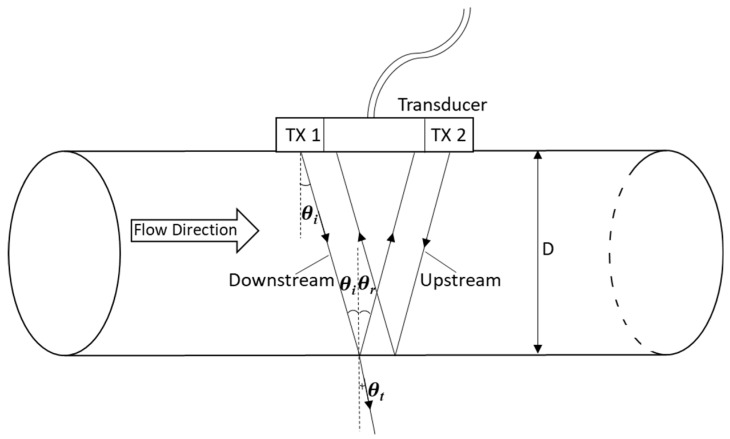
Illustration of pipe geometry and propagation and reflection of ultrasound waves.

When ultrasonic waves travel through the pipe and water and then impinge on the opposite side of the pipe, a portion of the incident acoustic energy is transmitted through the pipe, while the rest is reflected from the pipe, as shown in Figure 3. The transmission and reflection coefficients of acoustic energy were determined by applying Snell’s law with the ratio between acoustic impedance and incidence angle [24]. As there is nearly full reflection from the metal pipe, the incidence angle (*θ_i_*) is the same as the reflection angle (*θ_r_*). Considering the incidence and reflective angles, the matching receiver elements for the transmitter elements were calculated (e.g., matching elements for incidence angle (*θ_i_*) of 20.13° are 90 and 39 for the downstream and upstream, respectively).

**Figure 4:**
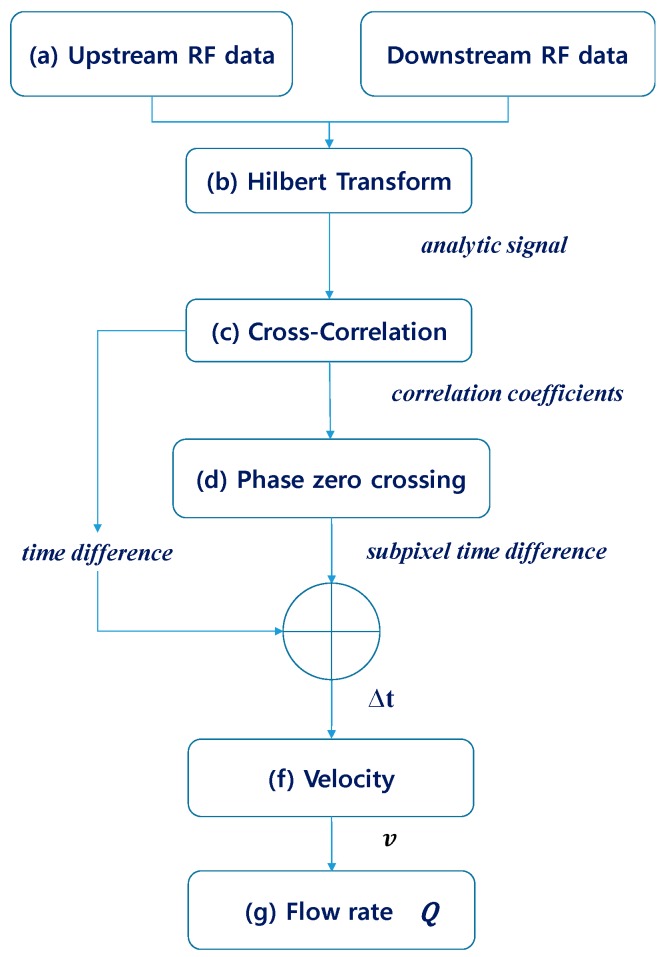
Flow estimation block diagram.

Most of the transmit-time ultrasonic flowmeters performed time estimation using the conventional zero-crossing approach, which detects the zero-crossing time of the received ultrasonic signals [5,25]. Afterward, the zero-crossing time difference between the upstream and downstream was measured. In this study, transit-time estimation was performed using the cross-correlation with phase zero crossing and the ultrasonic signals were interpolated by a factor of 100. Cross-correlation can estimate the time shift in the sampling interval. Then, in order to achieve subsample-level time estimation, the Hilbert transform phase zero-crossing method with linear interpolation was used [26,27]. The phase response of the cross-correlation in the Hilbert transform domain linearly crosses zero at the peak of the cross-correlation. Thus, the peak of the correlation in the subsample-level time could be estimated from the Hilbert transform phase zero-crossing. For the comparison of flow estimation methods, time-shift estimations were performed using both the conventional zero-crossing method and the cross-correlation with the phase zero-crossing method on the same dataset. The proposed estimation process in this study is shown in Figure 4.

The time difference between the upstream and downstream (Δt) can be described as

(1) $$\Delta t=t_{up}-t_{down}=\;\frac{2Lsin\theta }{c^{2}-V^{2}sin^{2}\theta }V_{beam},$$

where *L* is the ultrasonic path length through the fluid, *c* is the speed of sound in the fluid, *V_beam_* is the average flow velocity along the ultrasonic beam, and *θ* is the angle of the propagating ultrasonic wave. As liquid flow velocity is much lower than the speed of sound in general, the flow velocity equation was derived as follows:

(2) $$V_{beam}=\frac{c^{2}\Delta t}{2Lsin\theta }.$$

Therefore, the flow rate *Q* is given by

(3) $$Q=K\frac{\pi D^{2}}{4}V_{beam},$$

where *D* is the diameter of the pipe, and *K* is the correction factor resulting from the ratio of the actual average velocity in the pipe and the velocity measured along the ultrasonic beam. As the Reynolds number was in the range of 4 × 10^3^ to 3.8 × 10^4^, the flow in the pipe was turbulent where the correction factor depended on the Reynolds number, pipe radius, and pipe roughness. As the pipe roughness could not be measured, *K* (0.93~0.94 in this study) was theoretically calculated using the Reynolds number [13]. In addition, the speed of sound was affected by temperature. Thus, the liquid temperature (20–25 °C) was monitored in real-time while acquiring the data and reflected on the speed of sound in the estimation.

Because bubbles in the fluid can significantly affect the time estimation performance, bubble concentrations in the pipe were monitored using ultrasound imaging. Ultrasound images of the pipe cross-section (i.e., same view as the flow meter) were generated by transmitting plane waves using the 64 array elements (elements 1–64) placed on the pipe surface and collecting the received signals from 32 array elements (elements 33–64). No beamforming was applied for imaging. The imaging region measured 9.5 mm in width by 9.5 mm in height, and the center of the region was at 15 mm depth of the fluids in the pipe. Ultrasound images of the fluid were acquired at different flow rates. As bubbles are strong reflectors of ultrasound waves in the fluid owing to the high impedance mismatch between the fluid and air, the strength of the reflected ultrasound signals in the water was measured from the acquired ultrasound images. When the signal strength is higher, the concentration of the bubbles can be interpreted to be higher. Thus, the relative change in bubble concentration was measured by averaging the signal strength in the imaging region at different flow rates.

### 2.3. Angular Compensation

Angular compensation from various plane wave angles was applied to improve the accuracy of flow measurement using the linear array transducer. To control the incidence angle by delaying the transmission time of each element of the array transducer, multi-angle transmission was used. In this study, 24 incidence angles were applied for both upstream and downstream transmissions. Then, the estimated flow rates were averaged, which is a similar concept as the spatial averaging method [28]. To measure the time difference, each plane wave angle had appropriate matching receiver elements for the transmitter elements in the upstream and downstream. The positions of the receiver elements were calculated from the positions of the transmitter elements, incidence angle, and Snell’s law. Once the theoretical element matches were calculated from Snell’s law, the receiver elements were further selected by searching for the minimum shift at no flow near the theoretical elements. Table 1 lists the angle and appropriate center elements of the receiver for both upstream and downstream.

### 2.4. Analysis of the Measurements

To compare the performance of flow rate estimates, linear regression using the least-squares method was performed between the reference measurements using the electromagnetic flowmeter (i.e., predictor variable) and the measurements using various approaches in our study (i.e., response variable). In the linear regression, the slope (i.e., regression coefficient) and intercept values were calculated to determine the accuracy of the measurements (ideally, regression coefficient = 1 and intercept = 0). The coefficient of determination R^2^ was also evaluated to determine the goodness-of-fit.

## 3. Results

### 3.1. Ultrasound Data

**Figure 5:**
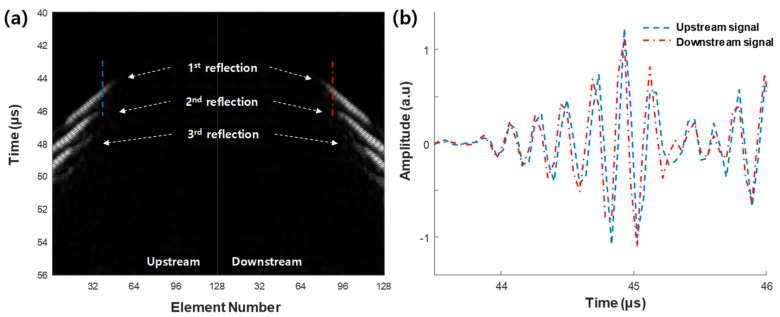
Ultrasonic (**a**) 2D images and (**b**) signals of upstream (blue dashed line) and downstream (red dash-dotted line) at a flow rate of 35 L/min. Dashed and dash-dotted lines in (**a**) are the positions where the ultrasonic signals in (**b**) are displayed. The arrows indicate the 1st, 2nd, and 3rd reflections.

Ultrasound data acquired from the array transducer at a flow rate of 35 L/min is shown in Figure 5. Figure 5a displays the data from receiver elements 1–128 for both upstream and downstream. In addition to the first reflection from the pipe surface, there were two more reflections. The ultrasonic signals from the upstream (dashed line) and downstream data (dash-dotted line) in Figure 5a are shown in Figure 5b. A time difference was observed between the upstream and downstream signals. As the velocity for the downstream was higher, the signal from downstream arrived faster.

### 3.2. Focused Beam vs. Plane Wave

**Figure 6:**
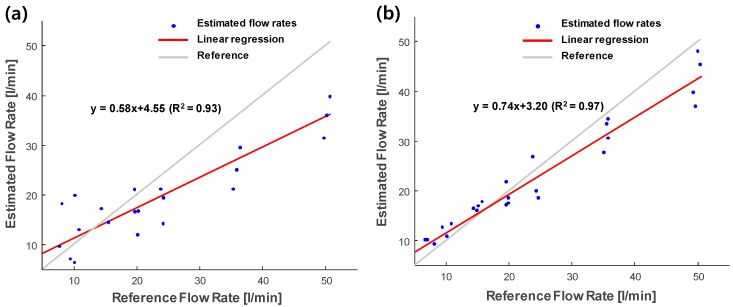
Estimated flow rates using (**a**) focused beam and (**b**) plane waves.

Figure 6 illustrates the flow rates measured by the ultrasonic flowmeter using the array transducer for focused beam (Figure 6a) and plane-wave beam (Figure 6b) compared with the flow rates measured using the electromagnetic flowmeter (i.e., reference). For each flow rate, four sample data were used (i.e., 4 × 8 = 32 total samples). Overall, the flow rates from both focused beams and plane waves were underestimated. The regression coefficient was 0.58 and the intercept was 4.55 for the focused beam, while the ultrasonic flowmeter using plane waves showed a higher regression coefficient (0.74) and a lower intercept (3.20). These results demonstrate that plane waves can estimate flow rates more accurately than the focused beam. Thus, plane waves were chosen for analysis.

### 3.3. Zero-Flow Calibration

**Figure 7:**
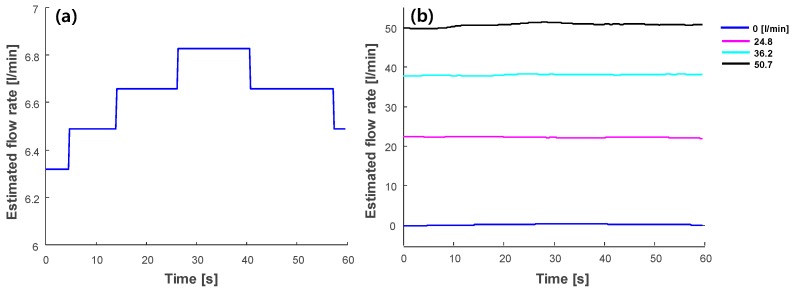
Estimated flows for (**a**) no flow, and (**b**) eight flow rates (reference flows at 0, 24.8, 36.2, and 50.7 L/min) after zero-flow calibration.

Ideally, the flowmeter should estimate the zero flow rate when there is no fluid flow in the pipe. However, the flow rate estimation can be affected by the properties of the pipe, the impedance matching of the transducer, and the flow condition. Hence, zero-flow calibration was applied by subtracting the estimated flow rate at no flow [5]. Ultrasound data using the plane wave approach was recorded at eight flow levels for 60 s with a frame rate of five frames per second (i.e., total 300 frames). The flow rates were estimated and zero-flow calibration was then applied. Figure 7 shows the estimated flow rate for no flow (Figure 7a) and for various flow rates (25, 35, and 50 L/min) after zero-flow calibration (Figure 7b). The mean and standard deviation of the zero flow were 6.64 L/min and 0.15, respectively. As shown in Figure 7b, the proposed flowmeter could perform stable measurements at various flows, which were measured for 60 s. The means and standard deviations of the estimated flow rates were 22.2 and 0.13, 38.0 and 0.16, and 50.6 and 0.44 L/min for the reference flow rates at 24.8, 36.2, and 50.7 L/min, respectively. As the flow rates increased, the standard deviations also increased. However, the estimated errors and deviations were significantly lower than the zero-compensation value (i.e., 6.64 L/min in this example). Thus, zero-flow calibration can effectively offset the dependence on the physical conditions of the pipe.

### 3.4. Bubble Monitoring

**Figure 8:**
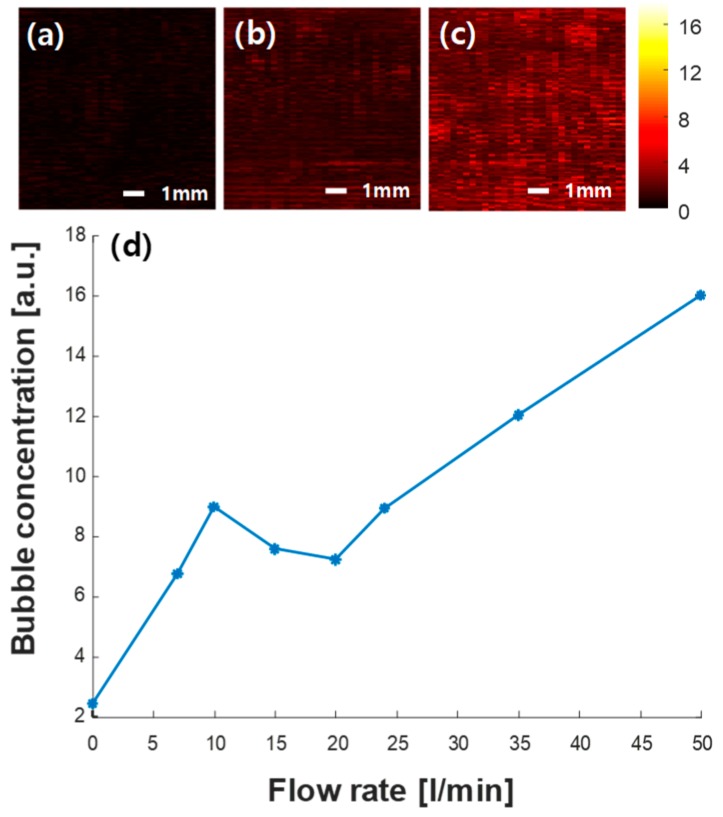
2D ultrasound images of the liquid flowing in the pipe (color bar represents the ultrasound signal strength) at flow rates of (**a**) no flow, (**b**) 25 L/min, (**c**) 50 L/min, and (**d**) the relative bubble concentrations at varying flow rates.

Figure 8a–c show the 2D ultrasound images of the fluid moving in the pipe at flow rates of 0, 25, and 50 L/min, respectively. The relative bubble concentration changes in the pipe, as shown in Figure 8d. Overall, bubble concentration increased gradually as the flow rate increased. Bubble concentrations at flow rates of 7, 10, and 15 L/min were relatively higher than those at the flow rate of 20 L/min as shown in Figure 8b. This can be attributed to the control of the valve leading to varying diameters of the pipe (i.e., narrower diameter to reduce flow) at flow rates lower than 20 L/min.

### 3.5. Flow Rate Estimation

**Figure 9:**
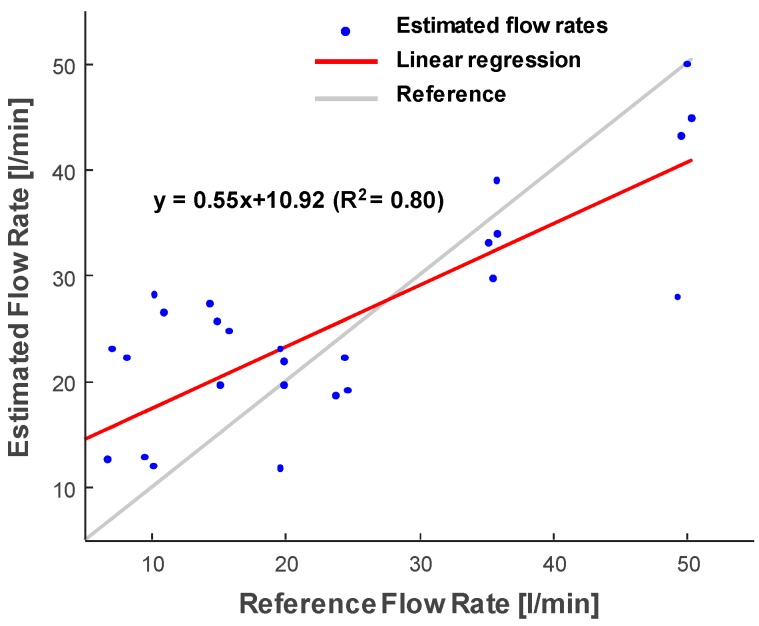
Estimated flow rates using the conventional zero-crossing method.

Figure 9 shows the measured flow rates using the conventional zero-crossing method for transit-time estimation. The regression coefficient and the intercept of the conventional zero-crossing method were 0.55 and 10.92, respectively. Compared to the result of the cross-correlation with the phase zero-crossing method (regression coefficient: 0.74 and intercept: 3.20) in Figure 6b, the estimates from the conventional zero-crossing method showed higher variations than the proposed method.

### 3.6. Multiple Angular Compensation

**Figure 10:**
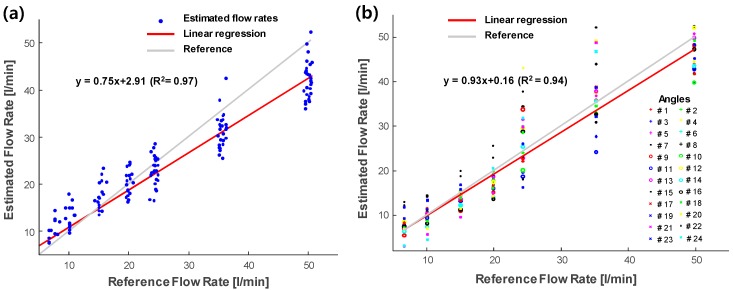
Estimated flow rates using (**a**) a single plane wave and (**b**) multiple angular compensation.

Figure 10 shows the flow estimation results of the ultrasonic flowmeter using a single plane wave (Figure 10a) and multiple angle plane waves (Figure 10b). In the case of a single plane wave, 138 data points with varying flow rates were used at a fixed incidence angle (angle #12 in Table 1). Compared to the results from 32 data points using a single plane wave (regression coefficient: 0.74 and intercept: 3.20) in Figure 6b, an improvement was hardly observed in the 138-data-point case (regression coefficient 0.75 and intercept: 2.91). Figure 10b shows the multiple plane wave case (regression coefficient: 0.93 and intercept: 0.16) with 24 angles (Table 1) applied for transmission. The results show that multiple angular compensation with spatial averaging can be utilized to achieve higher accuracy instead of simply averaging additional data points using a single plane wave.

## 4. Discussion

The current study demonstrated the feasibility of using the single-sided non-invasive ultrasonic liquid flowmeter with a linear array transducer. The ultrasound array transducer was used as the transmitter and receiver sensors. As shown in Figure 6, 58% and 74% of the flow rates of the electromagnetic flowmeter were measured by the focused beam and plane-wave methods, respectively. As the ideal focal position of the focused beam is on the surface of the opposite side of the pipe to receive a strong reflection from the pipe wall, the focused beam can have a high signal-to-noise ratio. However, in the non-invasive measurement, the beam experienced wave distortion when passing through the pipe and fluids, allowing the direction of the waves and the energy of the beam to be changed. Thus, the focused beam in this study had limited focusing. For the plane wave, the beam was broad; therefore, the effect from the wave distortion through the pipe was less. Thus, it was demonstrated that the plane wave can measure flow rate more accurately than the limited focused beam in non-invasive measurement. Although it would be complex to control the focused beam in the non-invasive case, perfect beam focusing can be achieved by precisely predicting the wave path in the pipe. Then, the focused beam will be further compared with the plane-wave method.

Moreover, it was shown that the accuracy of flow rate measurements using the ultrasound array transducer was lower than that of invasive reference measurements (i.e., electromagnetic flowmeter). The possible factors degrading the accuracy of our transit-time ultrasonic flowmeter were the small inner diameter and pipe roughness, as well as the velocity distribution in the pipe [11,13]. To increase measurement accuracy, a multipath approach was suggested in the previous study [16,28]. By using multipath rather than a single path, the increased sound path coverage can improve measurement accuracy. A previous study applied the spatial averaging method by utilizing a single element transducer for transmission and eight array elements for reception to spatially average the corresponding eight ultrasonic paths [28]. The results showed that the spatial averaging method using multiple ultrasound paths enhanced the accuracy of the ultrasonic flow meter. To maximize the multipath compensation, it is necessary to decide the sound paths covering a wide range in the pipe. However, there were geometric limitations in our study; therefore, multipath was selected as the angle-varying plane waves. By controlling the transmit delays for transmission wave propagation, the incidence angle can be adaptively modified without changing the physical position and angle of the sensors. As shown in Figure 10, multiple angular compensation from various plane wave angles (24 angles in Table 1) improved the accuracy of 93% of the reference measurements. By averaging the measurements from multiple angular compensation, we can achieve higher accuracy. The results showed the advantage of multiple angle transmission of the ultrasound array transducer, unattainable neither by wedge sensors with physical angles nor by the simple averaging of multiple measurements from a single angle. This study demonstrated the validity of the ultrasonic flowmeter using the array transducer by utilizing the transmit delays control and multiple angular compensation. Although 24 angles were used in this study owing to the limitation of our current data acquisition system, more ultrasonic paths (i.e., angles in this study) can improve the accuracy as demonstrated in the previous studies [16,28,29]. For the multipath approach, the velocity values can be integrated with weighting factors considering the geometrical positions of the sound paths [16,29]. In our study, multiple beams were not significantly different in the geometrical positions. Thus, the estimated velocities were spatially averaged without weighting factors.

As this study uses a flat linear array, the irregular condition of the pipe surface can disturb the incident waves and eventually lead to measurement errors. Thus, it is expected to have an even surface for at least the length of the flat linear array or that a special measuring window with an even surface can be designed. In a previous study, a Plexiglass viewing window was installed on the metal pipe for the linear array transducer to provide a regular condition of the pipe [21]. Although the transducer used in this study was originally designed for human study, the ultrasound wave could be transmitted well through the pipe with no observation of impedance mismatch problem. In addition, the correction factor *K* was calculated using theoretical calculation considering the Reynolds number only. The correction factor *K* can be further validated through an experiment quantifying flow rates, such as liquid collection and measurement methods, to consider other factors affecting *K* such as pipe radius and roughness [11,13,30].

For the transit-time ultrasonic flowmeter, the time-shift estimation method plays an important role in determining the quality of measurements. In this study, we performed experiments to compare two estimation methods, i.e., the conventional zero-crossing method and cross-correlation with phase zero-crossing method. As shown in Figure 6b and Figure 9, the cross-correlation approach performed 74% of the reference measurements compared to 55% of the conventional zero-crossing method measurements. A disadvantage of the conventional zero-crossing method is that it depends on the threshold value applied to the signal before calculating the shift [5,25]. Thus, it is susceptible to changes in signal amplitude, which leads to large variations of the estimated flow rate. Moreover, the cross-correlation approach was more robust against variations in signal amplitude. A previous study compared the simple cross-correlation method and the cross-correlation with the phase zero-crossing method in the frequency domain using Monte Carlo simulation for invasive pitch-catch ultrasonic flowmeter [7]. The results showed that the cross-correlation with phase zero-crossing method achieved superior signal-to-noise-ratio. Thus, the cross-correlation with phase zero-crossing method can be employed for valid and reliable flow rate measurements. To compensate for the possible error due to the low sampling frequency, the ultrasonic signals were interpolated by a factor of 100. An advantage of the cross-correlation with phase zero crossing is that it is less affected by the sampling frequency than the conventional zero crossing method [31]. Still, the accuracy and precision of the flowmeter could be significantly degraded owing to the errors in the estimation using signals acquired in the relatively low sampling frequency (i.e., four times the transmitting frequency in this study) [32]. Thus, a higher sampling rate with at least 10 times of the transmission frequency will be further implemented and the effect of the sampling rate on the accuracy of the measurement will also be investigated. To further improve measurements, zero-flow calibration was applied to the estimated flow rates to remove the bias values due to the variations in the pipe, sensors, and flow conditions. As shown in Figure 7, zero-flow calibration can reliably compensate for the flow rate estimates for long measurement periods.

For ultrasound flow measurements in the pipe, bubble concentration has a great impact, as bubbles are strong ultrasonic reflectors. At a higher pump speed, more bubbles were generated as shown in Figure 8. Overall, variations in the estimated flow rates were higher when the flow rate increased. To avoid bubble generation, an improved pipe system can be designed with a larger reservoir and pipe radius for a more stable liquid flow. In this study, the amount of bubbles was quantified by ultrasound images acquired using the array transducer, as shown in Figure 8. Thus, the reliability of the measurement could be predicted based on this information. As shown in Figure 8b, there was a local peak of bubble concentration in the low flow rates (<20 L/min). The effect of the bubbles in the low flow rates was prominent in the focused beam (Figure 6a) and in the conventional zero-crossing method (Figure 9). However, the plane wave case (Figure 6b) did not present a high variation in the low flow rate. This suggests that the plane wave may be much less affected by bubbles. However, further research is needed to understand the effect of bubbles on flow measurement using the plane wave.

Figure 5 shows that there were at least three strong reflections of the ultrasound signal due to the Lamb waves that propagated through the pipe wall [33,34]. Although we used only the first reflection of the signal for the time-shift estimation in this study, the second and third reflections can also be employed to add more traveling paths. Moreover, the 2D ultrasonic signals can be processed directly for the time-shift estimation instead of ultrasonic signal line processing. The error of the traditional non-invasive flowmeter is about ±2–5%, which is more accurate than the current study [11,35]. Our future research will focus on investigating the full utilization of the ultrasonic flowmeter functions with an array transducer for further improving accuracy and precision. As the current approach was evaluated to demonstrate its feasibility in the limited experimental environment, extensive testing will also be performed on multiple pipes at different temperatures.

## 5. Conclusions

In this study, the performance of a single-sided non-invasive ultrasonic flowmeter with an array transducer was evaluated. The experimental results of multiple angular compensation confirmed that our approach can be applied to increase the accuracy of the ultrasonic flowmeter using the linear array transducer. It can also help monitor liquid flow non-invasively with reliable measurement accuracy.

## Figures and Tables

**Table 1:** Angles of plane waves and corresponding receiver element numbers (#: number).

Number of Angles	Incidence Angle (°)	Center Element # of Upstream	Center Element # of Downstream
1	22.83	28	101
2	22.59	29	100
3	22.35	30	99
4	22.11	31	98
5	21.86	32	97
6	21.62	33	96
7	21.37	34	95
8	21.13	35	94
9	20.88	36	93
10	20.63	37	92
11	20.38	38	91
12	20.13	39	90
13	19.88	40	89
14	19.63	41	88
15	19.38	42	87
16	19.12	43	86
17	18.87	44	85
18	18.62	45	84
19	18.36	46	83
20	18.10	47	82
21	17.85	48	81
22	17.59	49	80
23	17.33	50	79
24	17.07	51	78

## References

[1] Miller R.W. (1983). Flow Measurement Engineering Handbook.

[2] Pasquale G.D., Graziani S., Pollicino A., Strazzeri S. (2015). A vortex-shedding flowmeter based on IPMCs. Smart Mater. Struct..

[3] Bevir M.K. (1970). The theory of induced voltage electromagnetic flowmeters. J. Fluid Mech..

[4] Lefebvre P.J., Durgin W.W. (1990). A Transient Electromagnetic Flowmeter and Calibration Facility. J. Fluids Eng..

[5] Hamouda A., Manck O., Hafiane M.L., Bouguechal N.E. (2016). An Enhanced Technique for Ultrasonic Flow Metering Featuring Very Low Jitter and Offset. Sensors.

[6] Tsukada K., Tsuzuki N., Kikura H. (2015). A Study of Air-coupled Ultrasonic Flowmeter Using Beam Focusing. Energy Procedia.

[7] Mandard E., Kouame D., Battault R., Remenieras J., Patat F. (2008). Methodology for developing a high-precision ultrasound flow meter and fluid velocity profile reconstruction. IEEE Trans. Ultrason. Ferroelectr. Freq. Control.

[8] Tsukada K., Kikura H. (2016). Flowrate Measurement on Metal Pipes by Air-coupled Ultrasound. Int. J. Comput. Methods Exp. Meas..

[9] Woodcock J.P. (1975). Development of the ultrasonic flowmeter. Ultrasound Med. Biol..

[10] Lynnworth L.C., Liu Y. (2006). Ultrasonic flowmeters: Half-century progress report, 1955–2005. Ultrasonics.

[11] Sanderson M.L., Yeung H. (2002). Guidelines for the use of ultrasonic non-invasive metering techniques. Flow Meas. Instrum..

[12] Mandard E., Kouame D., Battault R., Remenieras J., Patat F. Transit time ultrasonic flowmeter: Velocity profile estimation. Proceedings of the IEEE Ultrasonics Symposium.

[13] Zhang H., Guo C.W., Lin J. (2019). Effects of Velocity Profiles on Measuring Accuracy of Transit-Time Ultrasonic Flowmeter. Appl. Sci..

[14] Iooss B., Lhuillier C., Jeanneau H. (2002). Numerical simulation of transit-time ultrasonic flowmeters: Uncertainties due to flow profile and fluid turbulence. Ultrasonics.

[15] Terao M., Tanaka H., Tanaka Y. (2014). Easy-setup clamp-on ultrasonic flowmeter. Jpn. J. Appl. Phys..

[16] Rajita G., Mandal N. Review on transit time ultrasonic flowmeter. Proceedings of the 2016 2nd International Conference on Control, Instrumentation, Energy & Communication (CIEC).

[17] Mahadeva D.V., Baker R.C., Woodhouse J. (2009). Further Studies of the Accuracy of Clamp-on Transit-Time Ultrasonic Flowmeters for Liquids. IEEE Trans. Instrum. Meas..

[18] Driveklepp A. (2007). Ultrasound Examination of Steel Pipes.

[19] Kang L., Feeney A., Su R., Lines D., Jäger A., Wang H., Arnaudov Y., Ramadas S.N., Kupnik M., Dixon S. Two-dimensional flexural ultrasonic phased array for flow measurement. Proceedings of the 2017 IEEE International Ultrasonics Symposium (IUS).

[20] Jäger A., Unger A., Wang H., Arnaudov Y., Kang L., Su R., Lines D., Ramadas S.N., Dixon S., Kupnik M. Ultrasonic phased array for sound drift compensation in gas flow metering. Proceedings of the 2017 IEEE International Ultrasonics Symposium (IUS).

[21] Kikura H., Hayashida T., Ito D., Aritomi M., Mori M. Application of linear ultrasonic array transducer to two-phase flow measurements. Proceedings of the 6th International Symposium on Ultrasonic Doppler Methods for Fluid Mechanics and Fluid Engineering.

[22] Thomenius K.E. Evolution of ultrasound beamformers. Proceedings of the 1996 IEEE Ultrasonics Symposium. Proceedings.

[23] Park S., Kang H. (2016). Numerical Analysis on Cross-Shaped Array with Dynamic Transmit Focusing for Enhanced Volumetric Ultrasound Imaging. Appl. Sci..

[24] Nishino H., Masuda S., Yoshida K., Takahashi M., Hoshino H., Ogura Y., Kitagawa H., Kusumoto J., Kanaya A. (2008). Theoretical and Experimental Investigations of Transmission Coefficients of Longitudinal Waves through Metal Plates Immersed in Air for Uses of Air Coupled Ultrasounds. Mater. Trans..

[25] Texas-Instruments (2015). TDC1000 Ultrasonic Sensing Analog Front End (AFE) for Level Sensing, Flow Sensing, Concentration Sensing, and Proximity Sensing Applications.

[26] Cabot R. (1981). A note on the application of the Hilbert transform to time delay estimation. IEEE Trans. Acoust. Speech Signal Process..

[27] Lubinski M.A., Emelianov S.Y., Donnell M.O. (1999). Speckle tracking methods for ultrasonic elasticity imaging using short-time correlation. IEEE Trans. Ultrason. Ferroelectr. Freq. Control.

[28] Kang L., Feeney A., Su R., Lines D., Ramadas S.N., Rowlands G., Dixon S. (2019). Flow velocity measurement using a spatial averaging method with two-dimensional flexural ultrasonic array technology. Sensors.

[29] Tresch T., Gruber P., Staubli T. Comparison of integration methods for multipath acoustic discharge measurements. Proceedings of the IGHEM 2006 International Group of Hydraulic Efficiency Measurement.

[30] ISO 8316 (1987). Measurement of Liquid Flow in Closed Conduits—Method by Collection of the Liquid in a Volumetric Tank.

[31] Strunz T., Wiest A., Fleury A., Fröhlich T. Influence of turbulence on ultrasonic flow measurements. Proceedings of the 5th IGHEM Conference.

[32] Moazzeni T., Ma J., Jiang Y., Li N. (2011). Flow Rate Measurement in a High-Temperature, Radioactive, and Corrosive Environment. IEEE Trans. Instrum. Meas..

[33] Silva J., Lima A., Neto D. (2008). Fouling Detection Based on Parameter Estimation. Systems Structure and Control.

[34] Huang S., Wang S., Li W., Wang Q. (2016). The Propagation Characteristics of Ultrasonic Guided Waves in Plate and Pipe. Electromagnetic Ultrasonic Guided Waves.

[35] General-Electric (2011). DigitalFlow™ XMT868i Panametrics Liquid Flow Ultrasonic Transmitter Datasheet.

